# First Complete Genome Sequence of Piscine Orthoreovirus Variant 3 Infecting Coho Salmon (Oncorhynchus kisutch) Farmed in Southern Chile

**DOI:** 10.1128/genomeA.00484-18

**Published:** 2018-06-14

**Authors:** Harry Bohle, Patricio Bustos, Laura Leiva, Horst Grothusen, Esteban Navas, Alvaro Sandoval, Fernando Bustamante, Karen Montecinos, Alvaro Gaete, Marcos Mancilla

**Affiliations:** a Laboratorio de Diagnóstico y Biotecnología, ADL Diagnostic Chile SpA, Puerto Montt, Chile; b Departamento de Salud Animal, Subdirección de Acuicultura, Servicio Nacional de Pesca y Acuicultura, Victoria, Chile

## Abstract

We report here the complete genome of an isolate of piscine orthoreovirus variant 3 sequenced from a moribund coho salmon with jaundice that was reared in a seawater farm in southern Chile. The genome consists of 23,627 bp, including 10 segments that range from 1,052 bp (segment S4) to 4,014 bp (segment L1).

## GENOME ANNOUNCEMENT

Piscine orthoreoviruses (PRVs) are a group of emerging fish viruses associated with myocardial and skeletal muscle disease and circulatory disorders in seawater-reared salmonids. PRVs belong to the genus Orthoreovirus, which is in the family Reoviridae, with virus particles that have an icosahedral symmetry and are nonenveloped and nonfusogenic. The genome of PRV consists of 10 segments of double-stranded RNA (1). PRVs are ubiquitous and have been reported in all main farming countries with a particularly high incidence in salmonids. PRV-linked diseases negatively impact the salmon industry in Norway (2), Japan (3), and Chile (4, 5) by causing mortality. In Chile, PRVs were detected for the first time in Atlantic salmon (4), and their prevalence has been increasing. Therefore, epidemiological data related to PRVs need to be updated, especially after the identification of distinct genotypes.

The most prevalent variant is PRV-1, the etiological agent of heart and skeletal muscle inflammation (HSMI) in Atlantic salmon, Salmo salar (6), and coho salmon, Oncorhynchus kisutch (5). Recently, PRV-2 was recognized as the cause of erythrocytic inclusion body syndrome in coho salmon (3). Another PRV variant (PRV-Om) was initially related to HSMI-like disease and was suggested to be species specific for rainbow trout, Oncorhynchus mykiss (7). To maintain taxonomic consistency, PRV-Om was later renamed PRV-3 (8). In sum, three variants of PRV have been distinguished to date, with PRV-1 and PRV-3 being closely related to each other.

Specimens were collected by the Servicio Nacional de Pesca y Acuicultura (Sernapesca) in the region of Los Lagos in 2017. Total RNA samples from a moribund coho salmon and a healthy control were purified from heart and kidney tissues. The removal of host rRNA was accomplished with a Ribo-Zero kit. TruSeq libraries were prepared to be run on an Illumina HiSeq 2500 platform, and high-throughput sequencing was performed at Macrogen, Inc. (Seoul, South Korea). From a total of 55,292,189 reads, 101 bp in length, 5,201 paired-end reads were assigned to PRV-3 with an *N*_50_ value of 1,361 bp and 22× coverage. All reads were *de novo* assembled with SOAP*denovo* version 2.0. The selected viral contigs were reassembled and annotated with BLAST2go version 4.1 (Table 1). The resulting S1 sequence of PRV-3 was highly homologous to the ones previously reported for PRV isolated from rainbow trout in Norway (7) and coho salmon in Chile (5), named PRV-Om and genotype II, respectively.

**TABLE 1:** Features of the genome of the PRV-3 strain (isolate ADLPRV3)

Segment	Length (bp)	No. of coding sequences	GenBank accession no.
L1	4,014	1	MH229776
L2	3,970	1	MH229777
L3	3,973	1	MH229778
M1	2,372	1	MH229779
M2	2,190	1	MH229780
M3	2,450	1	MH229781
S1	1,124	2	MH229785
S2	1,348	1	MH229783
S3	1,134	1	MH229784
S4	1,052	1	MH229782

Although genomic data unequivocally demonstrated the presence of PRV-3 and viral loads were consistent with an active infection (cycle threshold values as low as 18.6), histopathological examinations revealed systemic lesions characteristic of an infection with Piscirickettsia salmonis. Furthermore, cardiac lesions pathognomonic of an infection with PRV were absent, which hampered the clinical interpretation of PRV detection. At this moment, we can only speculate on the role of PRV-3 in the pathogenesis of icterus, the main clinical finding in the index case, and the etiology of jaundice syndrome, an infectious disease of presumed viral origin (9).

This complete genome sequence of PRV-3 will contribute to a better understanding of the epidemiology of PRV and allow for improvements in diagnostics.

### Accession number(s).

The sequences of segments comprising the PRV-3 genome reported here were deposited at DDBJ/EMBL/GenBank under the accession numbers listed in Table 1. The versions described here are the first versions.
